# Loneliness and mental health among the elderly in Poland during the COVID-19 pandemic

**DOI:** 10.1186/s12889-021-12029-4

**Published:** 2021-11-02

**Authors:** Beata Dziedzic, Anna Idzik, Ewa Kobos, Zofia Sienkiewicz, Tomasz Kryczka, Wiesław Fidecki, Mariusz Wysokiński

**Affiliations:** 1 Department of Development of Nursing, Social and Medical Sciences, Faculty of Health Sciences, Medical University of Warsaw, Żwirki i Wigury 61, 02-091 Warszawa, Poland; 2 Department of Basic Nursing and Medical Teaching, Department of Development of Nursing, Faculty of Health Sciences, Medical University of Lublin, 20-081 Lublin, Poland

**Keywords:** Anxiety, Depression, Irritability, Loneliness, Older people 60 +, COVID-19, Social isolation

## Abstract

**Background:**

The unexpected changes caused by the COVID-19 pandemic related to the fear of developing the disease, and the need for social distancing and isolation have had an effect on people’s mental health. These drastic changes can result in the development of anxiety, depressive symptoms and sense of loneliness. Elderly and chronically ill individuals are at a particularly high risk of developing COVID-19, suffering severe illness and dying as a result of it.

**Aim of the study:**

The aim of the study was to assess the prevalence of anxiety, depressive symptoms, irritability and loneliness in the elderly aged 60 years and older as a group exposed to the negative impact of the COVID-19 pandemic, and to analyze the relationships between loneliness and mental health of the respondents and sociodemographic variables and chronic diseases.

**Materials and methods:**

The study was conducted in Poland among 221 individuals aged 60+. The study material was collected using a sociodemographic questionnaire, Hospital Anxiety and Depression Scale (HADS-M) and a revised University of California Los Angeles loneliness scale (R-UCLA). Women accounted for 47.51% and men for 52.49% of study participants; the mean age was 65.18 (SD = 4.06).

**Results:**

In total, according to HADS-M, depressive symptoms were present in 19.15% of the participants and borderline states in 14.18% of them. Based on R-UCLA, moderate and moderately high sense of loneliness was present in 58.83% of the participants. Sense of loneliness was significantly correlated with the prevalence of depressive symptoms (*p* < 0.001).

**Conclusions:**

In this study, one in five participants experienced anxiety and depressive symptoms. Two out of three participants experienced a moderate sense of loneliness. Individuals who displayed a higher level of loneliness also had a higher severity of anxiety level depressive symptoms and irritability. Elderly individuals should be under special care due to their high risk of experiencing physical and mental effects of the COVID-19 pandemic.

## Background

Depression manifests primarily with sadness, fatigue, disturbances of sleep, appetite and concentration, loss of interests and anhedonia [1]. These problems may increase particularly during the COVID-19 pandemic as a result of change in living conditions due to the need for isolation and social distancing [2–5]. Negative effects of quarantine in the form of mental health deterioration were also observed during earlier epidemics, despite the fact that it was necessary to reduce disease spread [6]. In addition, in the case of SARS CoV-2 infection and development of COVID-19, a more severe course of the disease and a higher mortality rate are observed among older people [7] and those with comorbidities such as respiratory illnesses, arterial hypertension, diabetes, cardiovascular diseases and chronic kidney disease [8, 9]. In line with earlier reports, high levels of depressive disorders and anxiety were observed among elderly individuals, which were often associated with physical health deterioration, decreased social support [10] and the presence of chronic diseases [11, 12]. In earlier studies, 10–60% of participants with chronic heart failure were observed to have symptoms of depression and 11–45% of the participants had symptoms of anxiety [13], while 35% of patients with type-2 diabetes were found to have depression [14]. According to the World Health Organization (WHO) data, more than 20% of people aged 60 and older suffered from mental and neurological disturbances. The most common mental health problems in the elderly population included depression (7%) and anxiety disorders (3.8%) [15]. Data for Europe indicated that among people aged over 65, 8.1% experienced moderate, medium and severe symptoms of depression. In Poland, in this age group, 9.9% of individuals had symptoms of moderate, medium and severe depression. The European Union countries with the highest prevalence of depression among the elderly included: Hungary (16.5%), Bulgaria (16.1%), Portugal (15.1), Romania (13.6%), France (10.2%) and Cyprus (9.9%). The lowest number of depression symptoms among elderly individuals were observed in Holland 0.0%, Norway 2.3%, Finland 2.5%, Denmark 4.3%, Malta 4.5%, Sweden 5.3% and the Czech Republic 5.5% [16].

In January 2020, WHO declared a public health emergency of international concern due to a SARS CoV-2 epidemic outbreak [17], which was first observed in December 2019 in Wuhan, China, after numerous cases of pneumonia of unknown cause were previously recorded [18]. The dynamics of the SARS-CoV-2 spread made many countries in the world, including Poland, implement restrictions on movement, including a national lockdown from 12 March to 3 May 2020 [19]. Sudden restrictions adversely affected people’s mental health and caused an increase in the number of cases of anxiety and depression [20], particularly among the elderly [21], who are considered the largest risk group [22]. Observations of the state of mental health in older age groups during the COVID-19 pandemic indicate a more severe experience of anxiety and depression [22, 23], and loneliness, which intensifies anxiety and depression [24]. Some authors present different results indicating a lower level of anxiety in elderly individuals compared to younger ones during the COVID-19 pandemic [25–27].

Loneliness is a negative, unpleasant feeling associated with a lack or insufficient level of social relations compared to one’s expectations [28, 29]. However, loneliness is not only associated with a simple lack of social interactions [29]. The experience of loneliness is a complex system, which is potentially determined by certain life circumstances such as chronic diseases [30], widowhood, divorce or retirement [31]. In addition, apart from the aspect of physical presence of another person, loneliness may be associated with emotional relations, i.e. absence of a relationship with another person [30]. Researchers concluded that loneliness is associated with depression and is a predisposing factor for its development [30, 32, 33] and the experience of loneliness by the older community increases problems with both mental and physical health [34], and elevates the risk of all-cause mortality in elderly individuals who live alone [35]. Depression among the elderly is difficult to define since older people may tend to describe their symptoms of depression in terms of loneliness [36]. Earlier reports point to loneliness as a serious public health problem due to its influence on the development of many chronic diseases among the elderly [37]. In a study conducted in the United Kingdom there were 7.7% severely or very severely lonely respondents aged 65+ and 38.3% moderately lonely ones [38]. A similar result was obtained in a study by Victor et al., in which 7% of elderly participants reported severe loneliness [39]. In a study by Theeke, the rate of loneliness among Americans aged over 60 years was 19.3% [40]. Data concerning Europe show that 7% of adults experience loneliness, most commonly in countries such as Hungary, the Czech Republic, Italy, Poland, France and Greece. The lowest rates are observed in Holland, Denmark, Finland, Germany and Sweden. Higher rates of loneliness are found among the elderly with poorer health, unfavourable economic situation and limited social contacts [41].

During the COVID-19 pandemic, additional factors affecting the level of loneliness were identified such as social isolation and fear of coronavirus. In a study by Gaeta and Brygdes, slightly more than half of the participants (50.1%) reported their sense of loneliness to rise during the COVID-19 pandemic outbreak [42]. Other authors also noted an adverse effect of the COVID-19 pandemic on the levels of loneliness among the older community [43, 44]. Yeung Shan Wong et al. compared the level of loneliness in a group of elderly individuals before and during the COVID-19 pandemic. They demonstrated a higher sense of loneliness after the outbreak of the pandemic [45].

Elderly individuals should be under special care due to their higher risk of morbidity and mortality during crisis situations [46]. Experts call for action in order to mitigate the effects of isolation introduced in many countries in order to prevent the spread of the SARS-CoV-2 virus that are a threat to the mental and physical health of elderly individuals [47].

The aim of the study was to assess the prevalence of anxiety, depressive symptoms, irritability and loneliness in the elderly aged 60 years and older as a group exposed to the negative impact of the COVID-19 pandemic, and to analyze the relationships between loneliness and mental health of the respondents and sociodemographic variables and chronic diseases.

We verified the following research hypotheses:

H1. Women over 60 years of age show greater loneliness and worse mental health during the COVID-19 pandemic compared to men.

H2. Married/civil partners over 60 years of age show less loneliness and better mental health during the COVID-19 pandemic compared to those unmarried (widow/widower, single).

H3. Individuals over 60 years of age who are in a good financial shape show better mental health during the COVID-19 pandemic.

H4. Chronically ill individuals over 60 years of age show greater loneliness and worse mental health during the COVID-19 pandemic.

H5. Lower self-rating of physical and mental health status in individuals > 60 years of age during COVID-19 pandemic is associated with greater loneliness and symptoms of anxiety and depression as measured using the HADS-M.

## Methods

### Participants

The study was conducted on 6–12 October 2020 in Poland during the second wave of the COVID-19 pandemic in a group of 221 individuals aged 60+ with a mean age of M = 65.18, SD = 4.06. Women accounted for 47.51% of the study group and men for 52.49%.

Since the declaration of the pandemic in Poland, measures have been introduced to help mitigate it, including border closure, mass event bans, school closure, and restrictions on movement and trade. At the end of the first wave of the pandemic, when there was a decrease in infections, the Polish government decided to introduce 4 stages of lifting the restrictions, with a division of counties into red, yellow and green zones from the beginning of June 2020. Unfortunately, there was a significant increase in infections from October 2020, and the entire Poland was declared a red zone (second wave of the pandemic). Schools and shopping malls were closed again, and movement restrictions were introduced [48]. Most people, including the elderly, stayed mainly at home.

The study was conducted by a worldwide research company (Kantar Poland) specialized in social research. The study was conducted using random quota sampling. Two-step random sample selection was conducted, in which localities (rural and urban) were first selected at random and then quota sampling was performed. The random quota sampling was applied based on the stratified random selection, so that the participants reflect the population of 60-plus individuals, considering the administrative division of the country into types of localities (village, city, taking into account the population size in the latter), gender, age, education. Also in this respect, the sample reflects the population of 60-plus inhabitants of Poland. We used the database of the research company responsible for the implementation of our study to recruit study participants. Those recruited for the study were asked by email whether they consented to participate in the study. The content of the questionnaire was given in Polish, the initial questions concerned the frequency of symptoms of anxiety, depression and irritability, followed by the feeling of loneliness. The questionnaire ended with socio-demographic questions. Then, after giving consent, each participant received a link via e-mail with the address to the website on a special internet portal, which is the property of the company conducting the study. Each answer given by a participant of the study was sent to the research company immediately after approval. The study was conducted in accordance with the general principles set out in the ICC/ESOMAR International Code, data protection requirements, other relevant provisions and national best research practices.

The study was conducted in accordance with ethical principles set out in the Declaration of Helsinki. Before completing the questionnaire, the participants were asked for their consent to answer questions regarding the issues covered in it (the request for consent was included in the instruction). All the answers were treated as strictly confidential, and the participants were guaranteed full anonymity. The subjects participants could withdraw their participation from the study at any point of completing the questionnaire. The study was approved by the Bioethics Committee at the Medical University of Warsaw (approval no AKBE/232/2020).

### Instruments

#### Socio-demographic questionnaire

The questionnaire was used to collect sociodemographic data of the study group such us age, gender, marital status, education, employment status, place of residence, the person(s) with whom the respondent currently resided and self-rating of one’s financial situation. In addition, the participants were asked whether they had been in home quarantine any time during the COVID-19 pandemic, were in home quarantine at the time of questionnaire completion, had had a SARS CoV-2 test, knew any person with a positive test for SARS CoV-2 and whether they knew anyone currently in home quarantine, and about the way in which they performed their job during the pandemic. The subjects participants were also asked for subjective self-rating of their physical and mental health during the pandemic and whether they had selected chronic conditions such as cardiovascular, respiratory or endocrinological diseases, cancer, kidney disease and mental disorders.

#### Hospital anxiety and depression scale (HADS-M)

In order to evaluate anxiety and depression, a Polish version developed by Majkowicz et al. of HADS-M was used [49], which is a modified version of the Hospital Anxiety and Depression Scale (HADS) developed by Zigmond and Snaith [50]. Determined by Djukanovic et al., validation values for HADS for the general population aged 65–80 years were a = 0.92 for anxiety and a = 0.88 for depression [51]. In this study, the internal consistency of HADS-M was a = 0.94 for total score, a = 0.90 for anxiety subscale, a = 0.89 for depression subscale and a = 0.88 for irritability subscale. HADS contains 2 independent subscales: anxiety and depression. HADS is composed of 16 questions in total, with each of them scoring from 0 to 3 points. The maximum score separately for anxiety (7 questions) and depression (7 questions) is 21 points. The following interpretation was adopted in line with the questionnaire key for the anxiety and depression subscales: no disorders: 0–7 points, borderline states: 8–10 points, disorders: 11–21 points. The HADS-M scale in the study by Majkowska et al. contains two statements (2 questions) assessing the level of irritation, with a total score of 6. The irritability subscale is interpreted as follows: 0–2 no disorders, 3 borderline states, 4–6 the presence of disorders.

HADS-M is intended for screening to draw attention to the mental health of the study group; however, its interpretation does not constitute a clinical diagnosis, but only serves to indicate the scale of the problem and refer individuals with mental problems to a specialist.

#### R-UCLA

In order to rate the level of loneliness we utilised the Polish version of the UCLA Loneliness Scale (UCLA LS) developed by Russel et al. [52], which was validated by Kwiatkowska et al. (R-UCLA) [53]. The accuracy of R-UCLA with regard to older individuals was confirmed in a study by Velarde-Mayol et al., in which Cronbach’s alpha was a = 0.95 [54]. In this study, the internal consistency of R-UCLA was a = 0.90 for total score, α = 0.88 for intimate others, α = 0.83 for social others and a = 0.60 for belonging and affiliation. The scale is composed of 20 statements and the respondents can select one out of 4 answers (1 = “I never feel this way”, 4 = “I often feel this way”). The total score is a sum of scores from 3 subscales: belonging and affiliation, intimate others and social others. The maximum possible score is 80 points. Based on the interpretation for the whole scale, 4 levels of loneliness were defined: high level of loneliness: 65–80 points; moderately high level of loneliness: 50–64 points; moderate level of loneliness: 35–49 points and low level of loneliness: 20–34 points [55].

### Statistical analyses

The normality of data distribution was determined using the Shapiro-Wilk test and homogeneity of variance was checked with the Levene’s test. Differences between groups were assessed with Kruskal-Wallis test with post-hoc Dunn’s test for more than two groups being compared. For two comparison groups, the following were used: Student’s t-test for independent samples, the Cochran–Cox’s test for an unmet condition of homogeneity of variance or Mann–Whitney U-test. Correlation analysis was performed using Pearson’s linear correlation coefficient r. Results for which the probability level met the condition *p* ≤ 0.05 were considered statistically significant. The calculations were performed using Statistica 10.0. (Poland).

## Results

During the COVID-19 pandemic, 3.62% of the participants stayed in home quarantine, while the remaining 96.38% did not. Testing for SARS CoV-2 was performed in 9.93% of the participants, with a positive result for 2.7% and a negative result for 7.23% of them. Among the participants, 18.55% knew someone with a positive result of a SARS CoV-2 test and 38.45% knew someone who stayed in home quarantine. Among working individuals, the majority did not work during the pandemic (65.52%), 19.46% performed their job at the usual workplace, 6.78% did remote work, and 7.23% worked both remotely and on-site (Table 1).

**Table 1:** Questions on COVID-19 pandemic

Parameter		N	%
Were you under home quarantine during the COVID-19 pandemic?	No	213	96.38
	Yes	8	3.62
Were you under home quarantine at the time of the survey?	No	202	91.40
	Yes	19	8.60
Have you been tested for SARS CoV-2 infection?	No	199	90.07
	Yes	12	9.93
Have you been tested positive for SARS CoV-2	No	16	7.23
	Yes	6	2.70
Do you know any people tested positive for SARS CoV-2 infection?	No	180	81.45
	Yes	41	18.55
Do you know any people who are currently under home quarantine?	No	136	61.55
	Yes	84	38.45
How did you work during the pandemic?	at the usual workplace	43	19.46
	remotely	15	6.78
	both remotely and on-site	16	7.23
	NA/ did not work	147	65.52

The study included 221 individuals aged 60+ with a mean age of 65.18, SD = 4.06 (r-Pearson’s = 0.79, *p* = 0.240 in HADS-M; r-Pearson’s = 0.03, *p* = 0.581 in R-UCLA).Women accounted for 47.51% of the study group and men for 52.49%. In the study group, 71.04% of the participants were in a relationship, the majority of the participants had secondary/post-secondary education (62.44%), and employed individuals accounted for 24.9%. In total, 84.62% of individuals came from urban areas and 15.38% from rural areas. The majority of the participants lived with a spouse/partner (57.46%). There were 5.43% of the participants who rated their financial situation as very good and 4.53% who rated it as very bad. Based on HADS-M, a higher level of depressive symptoms (*p* = 0.046) was observed among women and those individuals who rated their financial standing poorer (*p* = 0.010). As far as the level of loneliness as per R-UCLA is concerned, a lower level of loneliness (*p* = 0.002) was observed among individuals in relationships (37.22) compared to widows and widowers (42.76) and single individuals (40.72), while people who lived alone had a higher level of loneliness (42.29) than those living with a partner or family (*p* = 0.018) (Table 2).

**Table 2:** HADS-M and R-UCLA scores with regard to sociodemographic variables (*n* = 221)

Parameter	N	% of all participants	Depression		HADS-M t/H/r	Loneliness		R-UCLA t/H/r
			M	SD		M	SD	
**Gender**								
Female	116	47.51	14.11	10.01	t = 2.00 ***p*** **= 0.046**	39.39	10.28	t = 1.13 *p* = 0.258
Male	105	52.49	12.41	9.35		37.87	9.61	
**Marital status**								
In a relationship	157	71.04	13.22	9.45	H = 0.22 *p* = 0.894	37.22	9.30	H = 11.82 ***p*** **= 0.002**
Widow/widower	35	15.84	14.21	10.19		42.76	10.84	
Single	29	13.12	13.15	10.92		40.72	10.59	
**Education**								
Primary/vocational	36	16.28	14.42	9.06	H = 1.50 *p* = 0.472	40.94	9.72	H = 2.63 *p* = 0.268
Secondary/post-secondary	138	62.44	12.99	9.73		37.99	9.90	
Higher	47	21.27	13.36	10.32		38.91	10.31	
**Employment status**								
Old-age pensioner/incapacity pensioner	162	73.30	13.72	10.06		39.35	10.08	
Unemployed	4	1.80	11.60	10.10	H = 1.24 *p* = 0.535	39.42	12.58	H = 5.34 ***p*** **= 0.069**
Employed	55	24.90	11.72	7.87		35.38	8.57	
**Place of residence**								
Rural area	34	15.38	13.09	10.34	H = 0.14 *p* = 0.930	38.09	10.76	H = 1.26 *p* = 0.532
Town	107	57.91	13.12	9.33		38.03	9.08	
City	80	26.71	13.83	10.32		40.36	11.26	
**Person with whom one currently resides**								
Alone	50	21.73	11.81	10.12	H = 4.597 *p* = 0.100	42.29	10.74	H = 8.07 ***p*** **= 0.018**
With spouse/partner only	125	57.46	12.78	9.04		37.60	8.60	
With family (children, relatives)	46	20.81	21.02	7.38		37.17	11.60	
**Financial situation rating**								
Very good	12	5.43	7.92	7.69	H = −13.28 ***p*** **= 0.010**	34.58	9.63	H = 9.28 ***p*** **= 0.054**
Quite good	78	35.29	12.90	9.21		38.24	9.60	
Neither good nor bad	89	40.27	12.12	8.25		37.78	9.52	
Quite bad	32	14.48	17.53	13.05		41.81	10.45	
Very bad	10	4.53	21.22	9.23		46.44	12.08	

**r** – Pearson’s correlation coefficient, **t** – Student’s t-test, **H** – Kruskal–Wallis test; **p** – statistical significance

The participants were asked to provide subjective rating of their physical and mental health during the COVID-19 pandemic and to name any chronic diseases they may have. Individuals who reported deterioration of their physical health (*p* = 0.001) and mental health (p = 0.001) were characterized by a significantly higher level of depressive symptoms, anxiety and irritability (HADS-M). In the loneliness scale (R-UCLA), statistically significant differences were found only for mental health deterioration (p = 0.001). Also, higher level of depressive symptoms anxiety and irritability (HADS-M) and a higher level of loneliness (R-UCLA) were found in participants with endocrinological diseases (*p* = 0.013, *p* = 0.021, respectively), kidney disease (*p* = 0.037, *p* = 0.044, respectively) and mental disorders (p = 0.001, *p* = 0.004, respectively) (Table 3).

**Table 3:** HADS-M and R-UCLA scores with regard to health self-rating and chronic diseases during the COVID-19 pandemic

Parameter	N	%	Depression		HADS-M H/Z	Loneliness		R-UCLA H/Z
			M	SD		M	SD	
**Subjective rating of physical health during the pandemic**								
It has definitely improved	5	2.26	12.60	14.64	H = 23.68 ***p*** **= 0.001**	36.60	12.62	H = 8.57 *p* = 0.072
It has somewhat improved	15	6.79	13.87	10.36		37.33	8.99	
It has not changed	159	71.94	11.45	8.36		37.70	9.64	
It has somewhat deteriorated	39	17.65	19.85	10.76		43.00	10.56	
It has definitely deteriorated	3	1.36	25.00	13.00		40.00	11.78	
**Subjective rating of mental health during the pandemic**								
It has definitely improved	6	2.62	7.20	12.34	H = 63.55 ***p*** **= 0.001**	32.60	12.38	H = 23.21 ***p*** **= 0.001**
It has somewhat improved	7	3.27	15.75	10.74		41.25	7.81	
It has not changed	164	74.21	10.39	7.09		37.23	9.20	
It has somewhat deteriorated	38	17.19	23.61	8.50		43.16	10.68	
It has definitely deteriorated	6	2.71	34.20	12.36		55.20	3.56	
**Cardiovascular diseases**								
No	113	51.13	14.13	10.66	Z = 0.73 *p* = 0.462	38.81	10.37	Z = −0.08 *p* = 0.933
Yes	108	48.87	12.50	8.70		38.53	9.62	
**Respiratory illnesses**								
No	189	85.52	13.01	6.33	Z = − 0.86 *p* = 0.383	38.49	10.15	Z = − 0.86 *p* = 0.392
Yes	32	14.48	15.03	11.11		39.71	8.91	
**Endocrinological conditions**								
No	178	80.54	12.46	9.30	Z = −2.46 ***p*** **= 0.013**	38.02	10.03	Z = −2.29 *p* = **0.021**
Yes	43	19.46	16.81	10.72		41.35	9.36	
**Cancer**								
No	211	95.47	13.36	9.75	Z = 0.44 *p* = 0.659	38.97	10.04	Z = 0.88 *p* = 0.379
Yes	10	4.53	12.10	9.49		35.90	8.29	
**Kidney disease**								
No	211	95.02	13.12	9.63	Z = −2.09 ***p*** **= 0.037**	38.33	9.85	Z = − 2.00 ***p*** **= 0.044**
Yes	11	4.98	16.73	11.22		45.09	10.63	
**Mental disorders**								
No	214	96.83	12.71	9.16	Z = −3.69 ***p*** **= 0.001**	38.31	9.82	Z = −2.83 ***p*** **= 0.004**
Yes	7	3.17	31.29	9.83		49.57	8.30	

**H** – Kruskal-Wallis test; **Z** – Mann–Whitney U-test; **p** – statistical significance

On average on HADS-M, the respondents scored M = 13.30, SD = 9.72. On the anxiety subscale, the mean scores were M = 6.50, SD = 4.73, on the depression subscale M = 4.77, SD = 4.48, and on the irritability subscale M = 6.80, SD = 9.72. On the loneliness scale, the subjects respondents scored a mean of M = 38.67, SD = 9.98 (Table 4).

**Table 4:** Anxiety, depression and irritability on HADS-M; Sense of loneliness on R-UCLA

	Variable	M	Median	Min.	Max.	SD
**HADS-M**	Anxiety subscale	6.50	5.0	0.0	21.0	4.73
	Depression subscale	4.77	3.0	0.0	20.0	4.48
	Irritability subscale	2.04	2.0	0.0	6.0	1.60
	Total score	13.30	11.0	0.0	47.0	9.72
**R-UCLA**	Belonging and affiliation	9.27	9.00	5.00	17.00	2.68
	Intimate others	21.58	20.00	11.00	40.00	6.24
	Social others	7.82	7.00	5.00	16.00	2.75
	Total score	38.67	37.00	21.00	66.00	9.98

In total, on HADS-M, depressive symptoms were found in 19.15% of the participants and borderline states were found in 14.18% of the participants. On the anxiety subscale, symptoms were found in 21.27% and borderline states in 13.58% of the respondents; on the depression subscale, symptoms were demonstrated for 11.76% of the participants. The proportion of individuals with disorders on the irritability subscale was 24.43%. A low sense of loneliness was found in 40.72% of the respondents, moderate in 39.37%, moderately high in 19.46% and very high in 0.45% (Table 5).

**Table 5:** HADS-M and R-UCLA scores

Scale	Parameter		n	%
**HADS-M**	Anxiety subscale	No disorders	144	65.16
		Borderline states	30	13.58
		Disorders	47	21.27
	Depression subscale	No disorders	163	73.75
		Borderline states	32	14.48
		Disorders	26	11.76
	Irritability subscale	No disorders	147	66.52
		Borderline states	20	9.04
		Disorders	54	24.44
	Total score	No disorders	150	66.67
		Borderline states	29	14.18
		Disorders	42	19.15
**R-UCLA**	Total score	Low	90	40.72
		Moderate	87	39.37
		Moderately high	43	19.46
		High	1	0.45

Analysis revealed statistically significant positive correlations between R-UCLA and HADS-M scores. Increasing loneliness scores were accompanied by increasing anxiety level, depressive symptoms and irritability scores. The highest strength of effect was found between two overall indicators: the total R-UCLA score (r = 0.48) and HADS-M score for the depression subscale (r = 0.44) and the total HADS-M score (r = 0.44). The r values between the different subscales were diverse, ranging from weak associations (r = 0.29) to strong associations (r = 0.48). The results are presented in Table 6.

**Table 6:** Correlations between sense of loneliness and anxiety, depression and irritability

R-UCLA	HADS-M			
	Anxiety	Depression	Irritability	Total score
Intimate others	*r* = 0.42	*r* = 0.44	*r* = 0.43	*r* = 0.44
	***p*** **< 0.001**	***p*** **< 0.001**	***p*** **< 0.001**	***p*** **< 0.001**
Social others	*r* = 0.29	*r* = 0.40	*r* = 0.37	*r* = 0.35
	***p*** **< 0.001**	***p*** **< 0.001**	***p*** **< 0.001**	***p*** **< 0.001**
Belonging and affiliation	*r* = 0.30	*r* = 0.34	*r* = 0.32	*r* = 0.32
	***p*** **< 0.001**	***p*** **< 0.001**	***p*** **< 0.001**	***p*** **< 0.001**
Total score	*r* = −0.41	*r* = 0.48	*r* = 0.46	*r* = 0.46
	***p*** **< 0.001**	***p*** **< 0.001**	***p*** **< 0.001**	***p*** **< 0.001**

## Discussion

The aim of the study was to assess the prevalence of anxiety, depressive symptoms, irritability and loneliness in the elderly aged 60 years and older as a group exposed to the negative impact of the COVID-19 pandemic. It was conducted during the second wave of the pandemic (October 2020), when a sudden increase in the number of SARS-CoV-2 infections was observed in Poland (over 10,000 daily). There was also an alarming increase in the number of deaths, which was the highest among elderly individuals with comorbidities. Since the beginning of the pandemic in Poland, there have been nearly 1,600,000 infected individuals and nearly 45,000 people have died [48].

In our study, which included individuals aged 60+, a higher prevalence of anxiety and depressive symptoms was found compared to data for Poland from before the COVID-19 pandemic: 9.9% for the elderly, with the highest figure in Europe being 16.5% [16]. In our study, borderline states and anxiety symptoms were found in about a third of respondents in the anxiety subscale, while borderline states and depressive symptoms were found in in a quarter of respondents in the depression subscale. The use of the irritability subscale, where borderline states and disorders were found in slightly more than a third of respondents, was an additional element.

Recent literature review emphasized a high prevalence of depression and anxiety symptoms in the whole population during the COVID-19 pandemic [56–58] and among the elderly [2, 22–24]. Our results confirm the findings of a growing prevalence of depressive and anxiety disorders during the COVID-19 pandemic. Depressive symptoms were observed in 26.25% of the subjects and anxiety in 34.85%, while irritability occurred in 38.91% of the participants.

During the initial period of the COVID-19 pandemic, Roob et al. conducted a study among 7127 London inhabitants aged over 50 years (mean age 70.7; SD = 7.4). They observed deterioration of depression components according to HADS in 12.8% of study participants (7.8% of men and 17.3% of women) and anxiety in 12.3% of individuals (7.8% of men and 16.5% of women), which are lower results than the ones in the present study [2]. Other authors obtained higher results compared to ours. In China, 37.1% of elderly individuals experienced symptoms of anxiety and depression [22]. Wu et al. recorded the highest level of anxiety (57.8%) and depression (47.2%) among individuals aged ≥60 years [59]. Among 103 Greek individuals in a study by Parlapani et al., moderate to severe depressive symptoms were found in as many as 81.6% and symptoms of anxiety were observed in 84.5% of elderly individuals [23]. All the above-mentioned studies were conducted during the COVID-19 pandemic. These differences may be due to temporal differences with regard to the development of the pandemic and the scale of the problem. China was the first country in which the disease was detected and Greece was one of the countries with the highest incidence rate. Similar results to those obtained in our study were recorded by Nwachukwu et al. In their study, 26.4% of respondents aged over 60 had depression, while the proportion individuals displaying anxiety was lower compared to our findings: 23.3% [27]. According to the data from a study by Bäuerle et al., reported during the pandemic in age groups 55–64, 65–74 and ≥ 75, mild anxiety was present in 27.6, 23.3 and 23.7% of individuals, respectively; moderate anxiety in 6.8, 4.4 and 5.9%, respectively, and severe anxiety in 4.9, 2.7 and 1.7% of the subjects, respectively. Serious depressive symptoms were found in the same age groups in 11.1, 8.5 and 10.2% of the respondents, respectively [60]. In some of the studies quoted above for comparison, different scales were used than in our study (depression was measured with Patient Health Questionnaire-9 [PHQ-9] and anxiety with Generalized Anxiety Disorder 7-item [GAD-7]).

The most important risk factors for COVID-19-related mortality include advanced age [7, 61] and comorbidities [8, 9, 62], whose presence can also increase depression and anxiety [30]. Our findings show a significant correlation between the level of loneliness and the prevalence of depressive symptoms and the presence of certain chronic diseases (endocrinological diseases, kidney disease and mental disorders). In a study by Röhr et al., approximately one in three subjects participants (36.9%) felt in danger during the COVID-19 pandemic due to their pre-existing conditions [63]. Elran-Barak and Mozeikov conducted a study during the first month of the pandemic among adult residents of Israel with chronic diseases only. They observed deterioration of physical health in 47.2% and deterioration of mental health in 50.5% of the subjects participants [64]. In our study, we observed lower figures regarding physical and mental health rating during the pandemic. Approximately one in five subjects participants reported deterioration in their physical and mental health during the pandemic: 19.01% for physical health and 19.90% for mental health. However, it needs to be taken into account that our study included the population aged 60+ only.

This study demonstrated higher levels of anxiety in women. Other authors [2, 22, 23, 65, 66] also identified a higher severity of mental health problems in female participants during the COVID-19 pandemic. However, in a study conducted in China, Wu et al. observed a higher level of depression and anxiety among men [59].

Loneliness can affect people of any age [67] and depends on many different factors, including health-related and emotional ones, and those associated with widowhood, divorce and retirement [30, 31, 68]. It should also be emphasized that elderly individuals who previously did not complain of loneliness were forced to avoid contacts with other people during the COVID-19 pandemic due to the need for social isolation and protection against the disease [69]. In some studies, it was demonstrated that loneliness is more commonly observed among individuals aged over 80 years [70]: as many as 40–50% of the participants stated that they often felt lonely [71]. In other research projects, a decrease in the level of loneliness was reported after 70 [72] and 85 years of age [73].

The results of a previous study concerning Europe indicated that 7% of adults experience loneliness [41]. The level of loneliness in the population aged over 65 was assessed in the SHARE research project conducted in 12 European countries. The rate of loneliness felt most of the time ranged between 1% for Switzerland and 10% for Greece, while the figures for substantial loneliness ranged between 4% for Switzerland and 20% for Greece. In Southern European countries, the level of loneliness was higher than in Northern European countries [74]. According to the results of our study with regard to sense of loneliness during the COVID-19 pandemic, high and moderately high levels of loneliness were present in 19.1% of the respondents and a moderate level in 39.37% of the participants, with a mean of 38.67 (SD = 9.98).

An increase in the level of loneliness among the elderly during the COVID-19 pandemic was found in 54% of the participants in the study by Kotwal et al. However, this rate decreased as the time passed after the outbreak of the pandemic to 41% after 4–6 weeks and to 27% after 13–15 weeks. In that study, individuals who reported an increase in the sense of loneliness also more frequently reported an increase in depression from 9 to 62% and anxiety from 9 to 57% due to COVID-19 [24]. In another study conducted in Northern California, 36.0% of elderly individuals reported feeling lonely during the COVID-19 pandemic [42]. In addition, it was found that loneliness was associated with anxiety and social isolation. Shrira et al. also demonstrated that loneliness correlated positively with anxiety and depression, with the mean for depression being M = 2.28 ± 0.90) [75]. In a study by Groarke et al., which was conducted during the pandemic, 20.6% of individuals aged 55–64 and 3.3% of participants aged 65+ felt lonely [76]. German researchers obtained much lower scores on the sense of loneliness [63]: in their study, 13.1% of elderly individuals declared a feeling of loneliness. The authors point to a lack of increase in the level of loneliness during the pandemic among elderly individuals and lower figures compared to younger age groups assessed in their study. It needs to be noted that the study was conducted during the initial period of the pandemic. Similar to that study, McGinty et al. demonstrated a sense of loneliness in 13.8% of Americans aged over 55. Their study took place in April 2020 [77].

Research on the level of loneliness among elderly individuals in the initial period of the pandemic and later provides interesting information. According to a study by van Tilburg, as the pandemic progressed, the feeling of loneliness increased, particularly as per the emotional loneliness subscale [78]. Luchethin et al. demonstrated that elderly individuals had a lower level of loneliness compared to young age groups; however, it was only the elderly whose sense of loneliness increased after social distancing was introduced [43]. However, in a study by Emerson, approximately 43% of respondents aged 60+ reported a sense of loneliness during the COVID-19 pandemic; among them, 30.9% of individuals stated that they were more lonely than before the pandemic and only 3.5% rated their loneliness as lower than before. Loneliness was the highest in people who lived alone (54.9%); in individuals who lived with others, the percentage was 41.7% [79]. In our study, the highest loneliness scores were also found in those who lived alone (M = 42.29, SD = 10.94).

Our current findings and comparisons of our results to those of other authors regarding psychological well-being and loneliness prior to the COVID-19 pandemic can confirm that the pandemic is one of the risk factors for deterioration of the mental health of the elderly. According to our observations, the COVID-19 pandemic is associated with increased symptoms of anxiety, depression and sense of loneliness in this age group. These findings are relevant for the issue of mental health support for sensitive groups, including the elderly during the pandemic, which has lasted for already over a year. Elderly people experienced many limitations related to the need for isolation, reduction or complete cessation of social contacts in this period. Problems related to the fear contracting SARS-CoV-2 and COVID-19 have also occurred Furthermore, the elderly had difficult access to doctors, which resulted in inadequate control of chronic diseases. Most appointments were in the form of telephone consultation at that time [80].

## Conclusions

In this study, one in five participants were found to have anxiety and depressive symptoms. Individuals who were affected to a larger extent included women, unemployed individuals, people living alone, participants with a poorer self-rating of financial situation, those with a lower subjective health rating and individuals with certain chronic diseases. Two out of three participants experienced a moderate level of loneliness; among them, the majority were single individuals, those living alone, the unemployed, people who rate their financial standing and physical and mental health as poor, and individuals with certain chronic diseases. People aged 60+ who displayed a higher level of loneliness also had a higher severity of anxiety level, depression and irritability. It is important for elderly individuals to be under special care, since they are at a higher risk of physical and mental impact of the COVID-19 pandemic.

### Limitations of the study

Our study did have certain limitations. It included a small number of participants. The questionnaires were completed and sent back over the internet; for this reason, in some of the questions, there was a low response rate. In total, 258 questionnaires were received, of which 221 completely completed questionnaires were included in the analysis, while the remining 37 did not meet the inclusion criterion. Also, difficult access to computer in the group of elderly people, the lack of skills to use it, especially in rural areas, could have had an impact on the limited number of responses and underestimated results in this age group. In addition, the dissimilarities between the results of our study and those of other studies may be due to methodology in that different scales were used. Furthermore, the majority of studies with which we compared our results were conducted at the beginning of the pandemic, while the present study was conducted during the second wave of the pandemic. Furthermore, the selection of the sample did not take into account such characteristics as income and sexual orientation, which may also be a limitation of this study and affect in what ways the sample is not representative.

However, the study does have its strengths such as random sample selection. The structure of the study sample reflects Poland’s population structure with regard to gender, age, education, size of place of residence, and province; thus, the sample is representative for the analysed population.

## Data Availability

The datasets generated and/or analyzed during the current study are not publicly available due to confidentiality, but data is accessible from the corresponding author on reasonable request.
